# Mesenchymal Stem Cell Based Regenerative Treatment of the Knee: From Basic Science to Clinics

**DOI:** 10.1155/2019/7608718

**Published:** 2019-09-09

**Authors:** Johannes Zellner, Brian Johnstone, Frank Barry, Henning Madry

**Affiliations:** ^1^Department of Traumatology, Caritas Hospital St. Josef, Regensburg, Germany; ^2^Laboratory for Experimental Trauma Surgery, Department of Trauma Surgery, University Medical Centre Regensburg, Germany; ^3^Department of Orthopaedics and Rehabilitation, Oregon Health & Science University, Portland, Oregon, USA; ^4^Regenerative Medicine Institute, National University of Ireland Galway, Ireland; ^5^Center of Experimental Orthopaedics, Saarland University Medical Center and Saarland University, Homburg, Saar, Germany; ^6^Department of Orthopaedic Surgery, Saarland University Medical Center and Saarland University, Homburg, Saar, Germany

Patients routinely inquire about alternatives to total knee arthroplasty during daily clinical practice. Mesenchymal stem cells have been proposed for repair and regeneration of different tissue structures of the knee joint like articular cartilage, meniscus, tendons, and ligaments. However, up to now, only a limited amount of stem cell-based therapeutic strategies have been introduced in daily clinical practice. This special issue focuses on recent developments in the use of mesenchymal stem cells for repair and regeneration of knee structures and their potential for clinical translation.

This special issue includes six high-quality peer-reviewed articles that illustrate innovative strategies and novel ideas on the topic of regenerative treatment of knee tissue structures. These papers include descriptions of influencing factors and dosage of progenitor cells, biomaterials for various applications, and stem cell sources and their use for treatment of osteoarthritis and cartilage repair. Of course, it is not possible to adequately represent all facets of this broad and rapidly expanding research field. However, we hope that this special issue adds new perspectives to the current knowledge on regeneration of knee musculoskeletal structures as an alternative to total knee arthroplasty.

